# Dynamics of RecA-mediated repair of replication-dependent DNA breaks

**DOI:** 10.1083/jcb.201803020

**Published:** 2018-07-02

**Authors:** Vincent Amarh, Martin A. White, David R.F. Leach

**Affiliations:** Institute of Cell Biology, School of Biological Sciences, University of Edinburgh, Edinburgh, Scotland, UK

## Abstract

Chromosomal replication is the major source of spontaneous DNA double-strand breaks (DSBs) in living cells. Repair of these DSBs is essential for cell viability, and accuracy of repair is critical to avoid chromosomal rearrangements. Repair of replication-dependent DSBs occurs primarily by homologous recombination with a sister chromosome. However, this reaction has never been visualized at a defined chromosomal locus, so little is known about its spatial or temporal dynamics. Repair of a replication-independent DSB generated in *Escherichia coli* by a rare-cutting endonuclease leads to the formation of a bundle of RecA filaments. In this study, we show that in contrast, repair of a replication-dependent DSB involves a transient RecA focus localized in the central region of the cell in which the DNA is replicated. The recombining loci remain centrally located with restricted movement before segregating with little extension to the period of postreplicative sister-chromosome cohesion. The spatial and temporal efficiency of this reaction is remarkable.

## Abstract (precis)

Spontaneous DNA double-strand breaks form during DNA replication and are largely repaired by recombination with a sister chromosome. Using live-cell fluorescence imaging, Amarh et al. show that repair of a replication-dependent break is rapid, localized, and involves a transient RecA focus.

## Introduction

Duplicating the genome is a fundamental requirement of life. Although the DNA replication machinery is capable of doing so successfully, it occasionally encounters obstacles that lead to replication fork stalling (Cox et al., 2000). Stalled replication forks are potential sources of double-strand breaks (DSBs; Michel et al., 2004). These spontaneous DSBs can be generated as a consequence of replication forks colliding with DNA-bound proteins such as transcription complexes (Marnef et al., 2017). Spontaneous DSBs are also formed at DNA nicks and gaps encountered by progressing replication forks (Kuzminov, 2001). Because one unrepaired DSB can be a lethal event, DNA DSB repair (DSBR) plays a critical role in underpinning chromosomal replication. This importance of DSBR during chromosomal replication is predicted to increase in organisms with larger genomes because the probability of DSB formation is expected to increase proportionally with the length of replicated DNA. The numbers of DSBs detected in *Escherichia coli* and human cells confirm this prediction. It has recently been estimated that the replication forks that duplicate the 4.6-Mbp genome of *E. coli* have an 18% probability of breakage as estimated by the percentage of cells with broken forks detected in the absence of repair by homologous recombination (Sinha et al., 2018). In human cells with a genome size of 3.2 Gbp, it is estimated that ∼50 spontaneous DSBs are repaired per cell cycle during chromosomal replication (Vilenchik and Knudson, 2003). DSBs emanating from broken replication forks are one-ended and cannot be repaired by nonhomologous end joining. However, such DSBs are likely to be particularly well suited for repair by homologous recombination because there is no requirement for an extensive DNA homology search as the site of the DSB is in close proximity to the unbroken sister chromosome.

The dynamics of DSBR in bacteria have been studied previously using live-cell fluorescence imaging. In these studies, DSBs have been generated by the rare-cutting I-SceI endonuclease (Lesterlin et al., 2014; Badrinarayanan et al., 2015), DNA damage–inducing drugs (Kidane and Graumann, 2005), or UV irradiation (Renzette et al., 2005; Centore and Sandler, 2007). Notably, RecA bundles or thread-like structures were detected after DSB induction. These extended RecA structures were proposed to mediate the extensive DNA homology search that was required for repair of I-SceI–induced DSBs (Lesterlin et al., 2014; Badrinarayanan et al., 2015). We reasoned that RecA bundles might not be required during repair of a replication-dependent DSB if the repair was initiated during the period of postreplicative cohesion of sister chromosomes in which an extensive DNA homology search is not required.

In this study, we investigated the spatial and temporal dynamics of RecA during the repair of a replication-dependent DSB in *E. coli*. We addressed this question by inducing a replication-dependent break in the *lacZ* gene, which is located on the right arm of the chromosome, approximately halfway between the origin and the terminus. Our study addressed the following questions: How long is a fluorescent derivative of RecA visible as a focus at the site of the DSB? Does the RecA focus at the site of the DSB mature to form a RecA bundle? Where is the RecA focus located in the cell, and how does its position relate to the localization of the DNA replication machinery? Does DSBR affect the duration of postreplicative cohesion at the *lacZ* locus?

We show that RecA works fast and does so in the center of the cell, close to where the break was formed during replication. No RecA bundle is observed, indicating that formation of this extended structure is not a necessary consequence of DNA breakage. The whole reaction (from breakage to separation of the recombining loci) is remarkably efficient as expected if the cell has evolved a mechanism to permit the repair of replication-dependent breaks with minimal perturbation to DNA replication.

## Results and discussion

### System for visualizing replication-dependent DSBR at the *E. coli lacZ* locus

**Figure 1.**
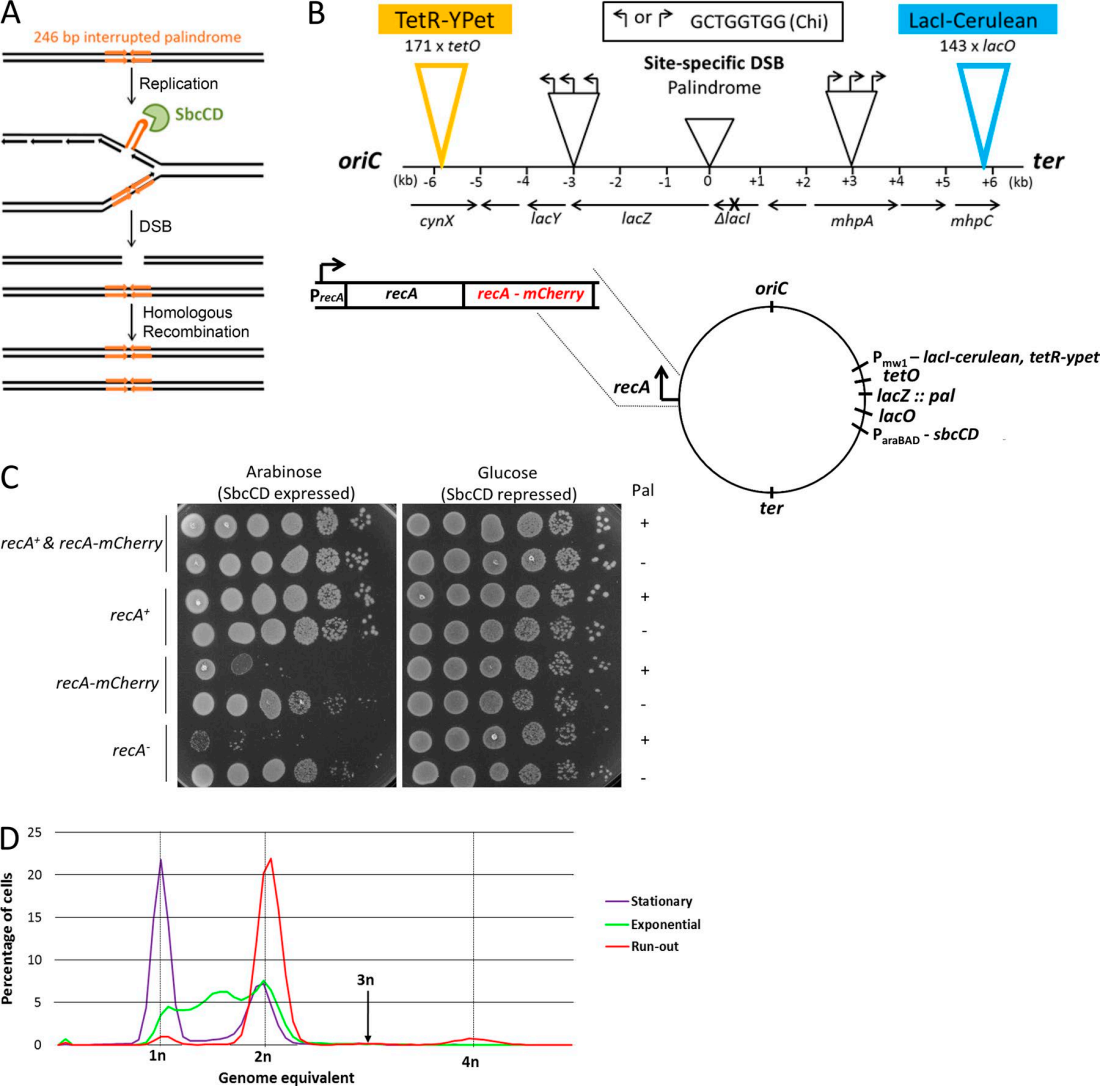
**System for visualizing DSBR at the *lacZ* locus. (A)** Schematic representation of replication-dependent DSB formation at the *E. coli lacZ* locus. **(B)** Schematic representation of the construct used for visualizing the site of the DSB at the *lacZ* locus. The location of the palindrome at the lacZ locus is indicated as 0 kb. The arrays of three Chi sites are represented by three arrows. The arrays of 171 *tetO* and 143 *lacO* sites are shown in yellow and blue, respectively. The precise number of operator sites in these constructs was determined by DNA sequencing with the primers listed in Table S3. A codon-diversified *recA-mCherry* gene was inserted in tandem with the endogenous *recA* gene at the native chromosomal locus. The promoter for *sbcC* and *sbcD* genes was replaced by the arabinose-inducible promoter, P*_araBAD_*. The *tetR-YPet* and *lacI-Cerulean* genes were expressed from a strong, constitutive, synthetic promoter at the chromosomal *ykgC* locus. **(C)** Spot-test assay showing that addition of the *recA-mCherry* gene does not affect cell viability after DSB induction at the *lacZ* locus. **(D)** DNA content of cells grown in M9–glycerol medium. The experiment was performed in triplicate. Representative data from one experiment are shown.

Live-cell fluorescence imaging was used to investigate the dynamics of repair of a site-specific DSB whose formation was dependent on chromosomal replication. The DSB was generated at the *lacZ* locus of the *E. coli* chromosome by SbcCD-mediated cleavage of a DNA hairpin structure formed by a 246-bp, interrupted palindrome on the lagging-strand DNA template during replication (Fig. 1 A; Eykelenboom et al., 2008). Arrays of *lac* and *tet* operator sequences were inserted on either side of the DSB site (White et al., 2008) and were preceded by arrays of three Chi sites (Fig. 1 B) to stimulate RecA loading by the RecBCD enzyme (Cockram et al., 2015). The *tet* and *lac* repressor genes were coupled to YPet and Cerulean genes, respectively, and inserted in tandem at the *ykgC* chromosomal locus, with these genes under the control of a strong constitutive synthetic promoter, P_mw1_. The P_mw1_ promoter was derived from the *ftsk* gene promoter (Wang et al., 2005) by mutating the −10 and −35 elements to their respective consensus sequences using site-directed mutagenesis. Binding of TetR-YPet and LacI-Cerulean proteins to the operator arrays generated coincident fluorescent foci, which marked the cellular location of the *lacZ* locus of the chromosome. Visualization of RecA was achieved by inserting a codon-diversified *recA-mCherry* gene in tandem with the endogenous *recA* gene, with both *recA* alleles under the control of the native promoter (Fig. 1 B). Codon diversification was performed to limit runs of homology between the *recA* alleles to <14 bp to minimize the probability of recombination between the two genes. Finally, the promoter of the *sbcD* and *sbcC* genes was replaced by the arabinose-inducible promoter (P*_araBAD_*) to enable induction of expression of the SbcCD protein, which is responsible for DNA hairpin cleavage, to generate the DSB at the *lacZ* locus (Eykelenboom et al., 2008). The tandem insertion of *recA-mCherry* and *recA* genes was constructed because the RecA-mCherry protein is only partially active, but *recA-mCherry* is recessive, and the partial diploid strain containing *recA-mCherry* and *recA* is fully recombination proficient, despite the incorporation of RecA-mCherry proteins into mixed filaments. Fig. 1 C shows that the partial diploid strain did not suffer any detectable loss of viability in response to DSB formation using our inducible system, confirming that the combination of RecA-mCherry and RecA conferred recombination proficiency. For the slow growth condition (M9 salt medium supplemented with glycerol) that was used in this study, cells had a single replicating chromosome in the absence of SbcCD expression (Fig. 1 D). It was therefore expected that only one DSBR reaction would occur per cell cycle when expression of SbcCD was induced in the palindrome-containing strain.

### RecA forms transient foci at the DNA DSB site

**Figure 2.**
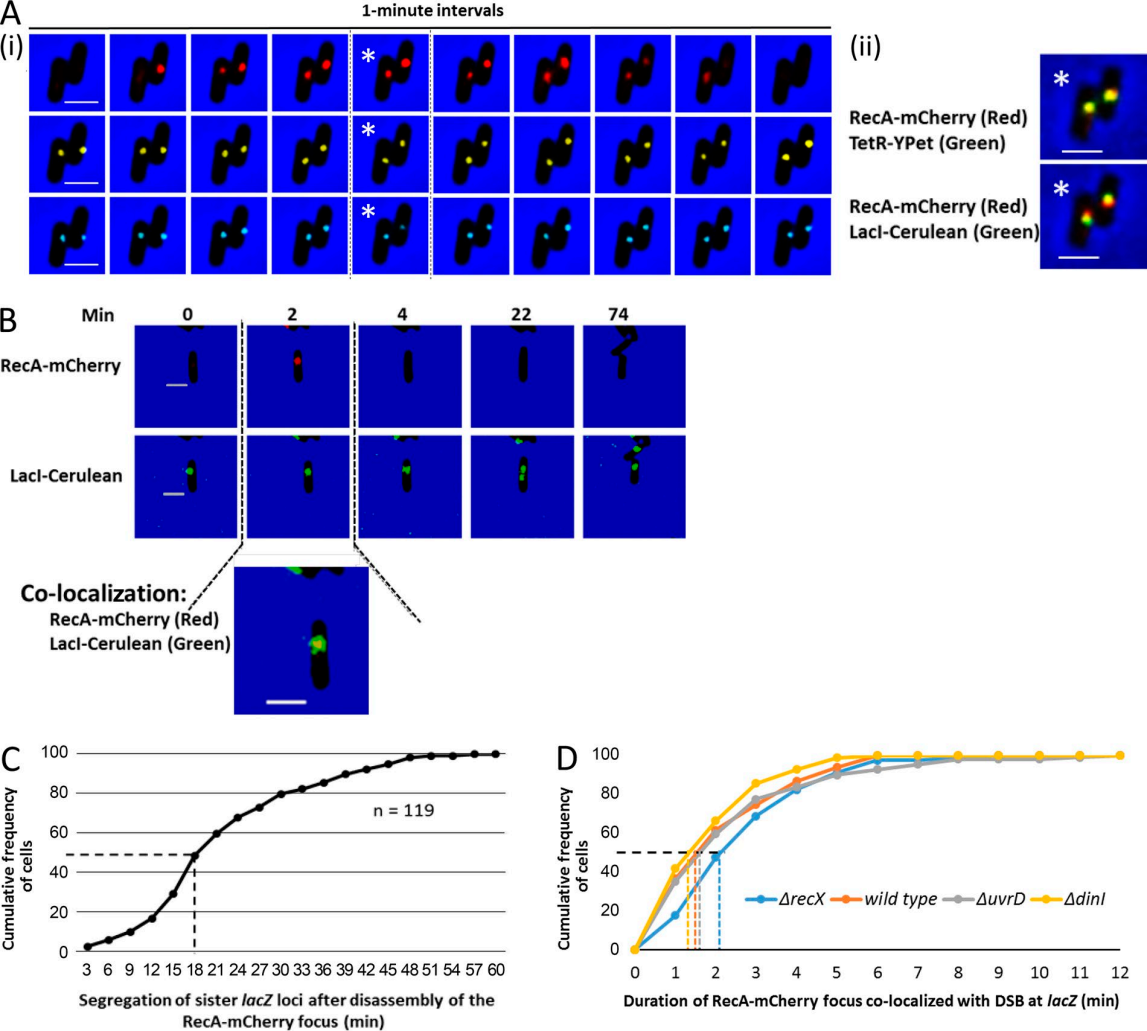
**Formation of a reversible and transient RecA focus at the *lacZ* locus during DSBR. (A)** Time-lapse fluorescence imaging of RecA-mCherry and the site of the replication-dependent DSB at the *lacZ* locus. The *lacZ* locus was visualized by TetR-YPet and LacI-Cerulean proteins bound to the *tetO* and *lacO* arrays, respectively. (i) Red, yellow, and blue spots represent RecA-mCherry, TetR-YPet, and LacI-Cerulean foci, respectively. (ii) Colocalization of RecA-mCherry focus with the LacI-Cerulean and TetR-YPet foci. Asterisks indicate the images used for the colocalization in ii. **(B)** Time-lapse fluorescence imaging of RecA-mCherry and the site of the DSB during repair, segregation, and cell division. Bars, 2 µm. **(C)** Duration of cohesion of sister *lacZ* loci after disassembly of the RecA-mCherry focus at the site of the DSB. n is the number of cells analyzed. **(D)** Duration of the RecA-mCherry focus at the site of the DSB WT cells and in *recX*, *dinI*, and *uvrD* mutants. Time-lapse imaging was performed at 1-min intervals for each strain after induction of SbcCD expression in the palindrome-containing strains. Dash lines represent the extrapolation of the median duration of the RecA-mCherry focus at the site of the DSB. Median durations for each of the mutants were not significantly different from the WT (P > 0.05) using the Mood’s median test. At least 80 cells were analyzed for the WT and each mutant strain.

After induction of SbcCD expression in the palindrome-containing strain, RecA-mCherry proteins were assembled to form a distinct focus, which colocalized with both the TetR-YPet and LacI-Cerulean foci, the fluorescent markers for the DSB site (Fig. 2 A; upper limit for colocalization is 0.4 µm). Subsequently, the RecA-mCherry focus disassembled to background fluorescence within the cell. Disassembly of the RecA-mCherry focus was followed by segregation of the sister *lacZ* loci into the opposite cell halves and, eventually, cell division (Fig. 2 B). The median duration between disassembly of the RecA-mCherry focus and segregation of sister *lacZ* loci was 18 min (Fig. 2 C).

Previous studies have suggested that the RecA nucleoprotein filament is stabilized by the DinI protein and is destabilized by the RecX and UvrD proteins in vitro (Stohl et al., 2003; Drees et al., 2004; Lusetti et al., 2004b; Veaute et al., 2005; Cox, 2007). In this study, we investigated the effect of Δ*uvrD*, Δ*recX*, or Δ*dinI* mutation on the duration of RecA-mCherry focus at the DSB site. Our data revealed that the median duration of the RecA-mCherry focus was 1.5 min in WT cells, 1.3 min in the Δ*dinI* mutant, and 2.1 min in the Δ*recX* mutant (Fig. 2 D). The effects of the Δ*dinI* and Δ*recX* mutations were small and not statistically significant. However, they were as expected for antagonistic roles of the RecX and DinI proteins on the stability of the RecA nucleoprotein filament (Lusetti et al., 2004a). Deletion of the *uvrD* gene caused a very minimal, statistically insignificant change to the median duration of the RecA-mCherry focus (median duration was 1.6 min), although some longer-lasting (4–6 min) foci were observed (Fig. 2 D). The RecA-mCherry foci colocalizing with the TetR-YPet and LacI-Cerulean foci were inferred to define RecA participating in DSBR at the site of the SbcCD-mediated cleavage of the DNA palindrome inserted in the *lacZ* gene. The transient duration of these RecA-mCherry foci suggest that the homology search and strand exchange are rapid events during repair of a replication-dependent DSB. This compares with periods on the order of 1 h after I-SceI cleavage, where RecA bundle formation to focus pairing takes a mean of 47 min, and bundles disassemble after a further 17 min (Lesterlin et al., 2014). Bundles or thread-like structures of RecA-mCherry were not detected in cells undergoing DSBR at the *lacZ* locus.

### DSBR localizes the *lacZ* locus to the midcell region

**Figure 3.**
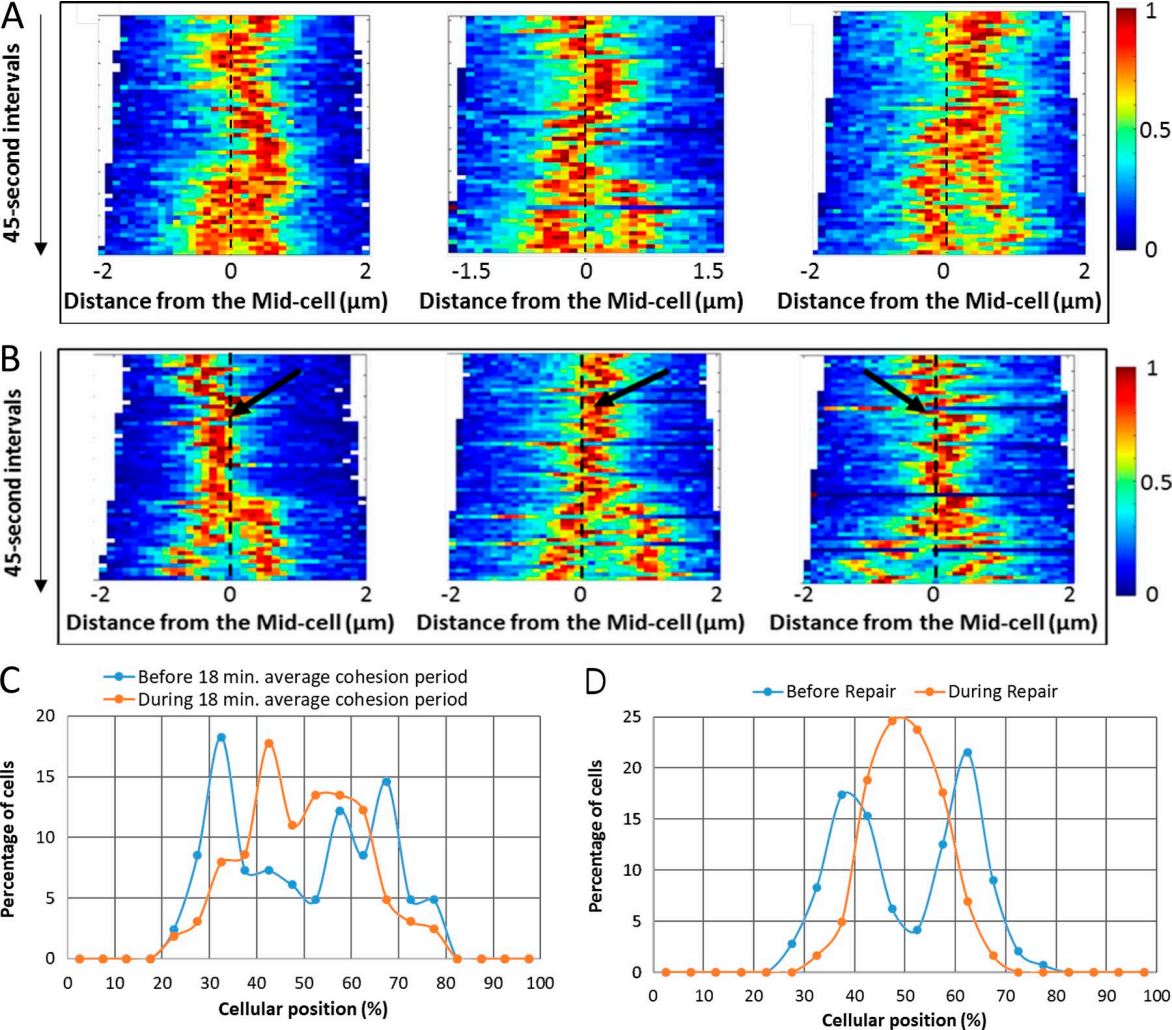
**Effect of DSBR on localization of the *lacZ* locus. (A)** Spatial dynamics of the *lacZ* locus in the absence of DSB induction at the *lacZ* locus. Dashed lines represent the midcell. Each kymograph shows a representative cell and was compiled from the phase-contrast and LacI-Cerulean fluorescence images of a cell acquired at 45-s intervals. **(B)** Spatial dynamics of the *lacZ* locus in the presence of DSB induction at the *lacZ* locus. Dash lines represent the midcell. Black arrows indicate the formation of the RecA-mCherry focus at the site of the DSB. Each kymograph shows a representative cell and was compiled from the phase-contrast and LacI-Cerulean fluorescence images of a cell acquired at 45-s intervals. **(C)** Localization of LacI-Cerulean foci, before foci splitting, in the absence of DSB induction at the *lacZ* locus. The data were separated into foci visible in the period before the last 18 min before foci splitting and foci visible during the last 18 min before foci splitting. The first period is expected to comprise mostly foci before DNA replication and replicative cohesion. The second period is expected to comprise mainly foci after DNA replication, during the period of postreplicative cohesion. *n* = 82 for the before-18-min mean cohesion period; *n* = 163 for the during-18-min mean cohesion period. **(D)** Localization of LacI-Cerulean foci, before foci splitting, in the presence of DSB induction at the *lacZ* locus. The formation of a RecA-mCherry focus at the DSB site was used as an indicator for repair (*n* = 144 before repair and *n* = 244 during repair). Heat maps are shown as colored bars in A and B, where 0 represents the background fluorescence within the cell, and 1 represents the maximum fluorescence of the LacI-Cerulean foci.

The effect of DSBR on the spatial localization of the *lacZ* locus was also investigated in strains containing, or not containing, the interrupted palindrome at the *lacZ* locus. After SbcCD expression in the strain that did not contain the interrupted palindrome, the *lacZ* locus exhibited dynamic movement, primarily on one side of the midcell, until segregation of the sister loci (Fig. 3 A). After segregation, one locus stayed close to the midcell, whereas the other migrated further, consistent with the left–right–left–right symmetry of the chromosome arms (White et al., 2008). In the palindrome-containing strain, the dynamic movement of the *lacZ* locus, which was also primarily on one side of the midcell became constrained in the vicinity of the midcell during the formation and after disassembly of the RecA-mCherry focus at the site of the DSB (Fig. 3 B). Distances of centroids of *lacZ* foci (before segregation) to an arbitrary cell pole are shown in Fig. 3 (C and D) and Fig. S1, whereas distances to midcell are shown in Fig. S2. Collectively, these data demonstrate that the central location of *lacZ*, from RecA-mCherry focus formation until segregation of the sister *lacZ* loci, is distinct from the locations of *lacZ* before DSBR and during replicative cohesion in cells not undergoing DSBR.

### DSBR occurs close to the site of *lacZ* replication

To investigate the relationship between DSBR and DNA replication, the *dnaN* gene encoding the β-sliding clamp of the replisome was tagged with the fluorescent protein YPet. Previously, fluorescent foci formed by YPet-DnaN proteins have been shown to be indicative of the cellular location of the chromosomal replisome (Wang et al., 2011). Time-lapse imaging confirmed that molecules of YPet-DnaN were assembled from background fluorescence to form a distinct focus in newborn cells, which was later disassembled before cell division (Fig. S3 A). Time-lapse imaging of the YPet-DnaN protein revealed that cells had either one or two YPet-DnaN foci during chromosomal replication. Interestingly, 28 out of 41 cells exhibited a single, predominant YPet-DnaN focus during the replication cycle, and this focus localized at or near the midcell (Fig. S3, A and B). In these cells, the single YPet-DnaN focus occasionally underwent transient separation to generate two foci, which were very close to each other at the midcell (Fig. S3, A and B). In the remaining 13 cells, the two YPet-DnaN foci that were generated by spatial separation of the sister replisomes were longer lived (Fig. S3 C). These observations suggest that, although the replisomes that duplicate each arm of the circular *E. coli* chromosome usually coexist in the vicinity of the midcell (Mangiameli et al., 2017), there is no requirement for them to do so to complete the cell cycle (Reyes-Lamothe et al., 2008).

**Figure 4.**
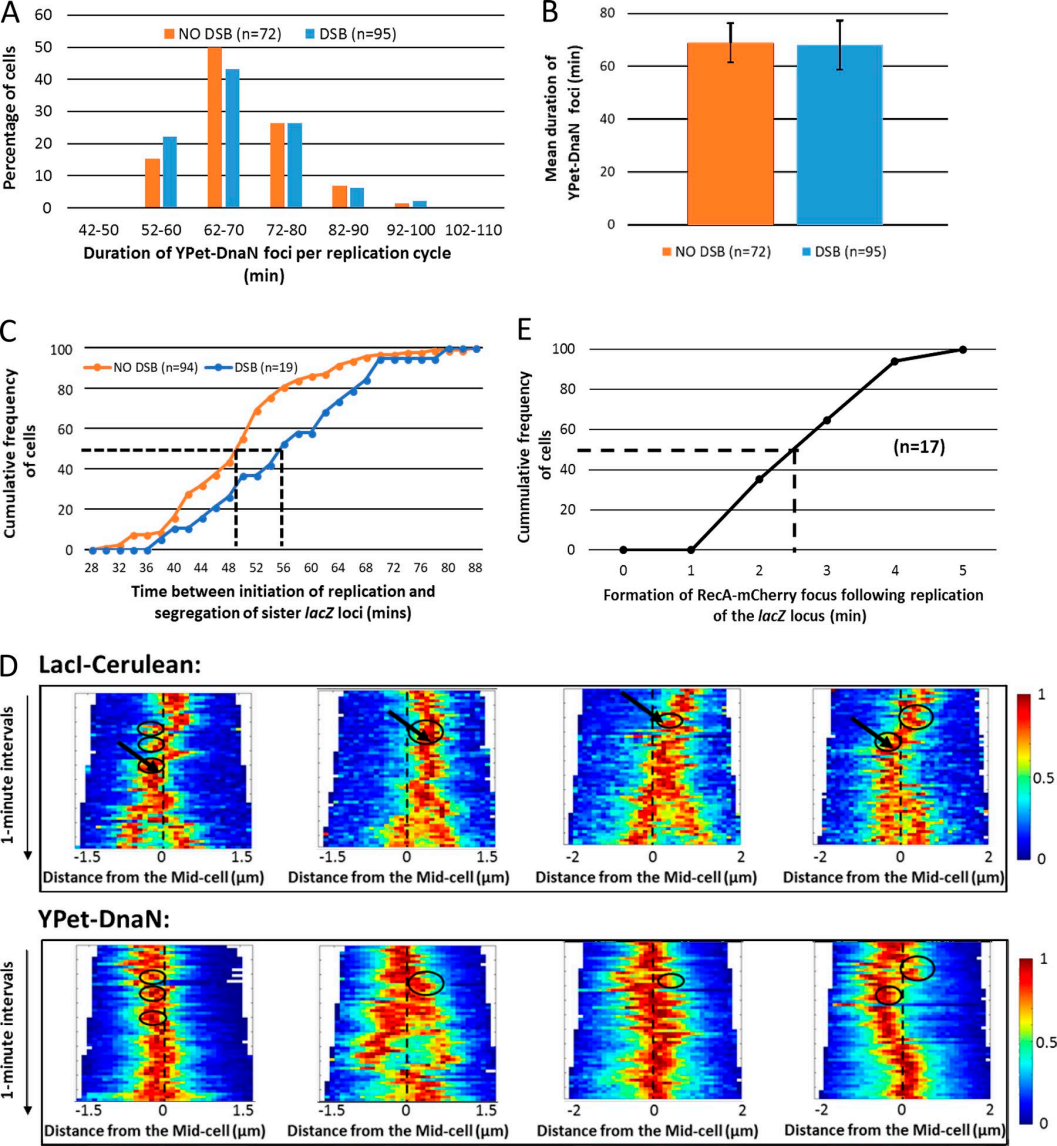
**Localization of the replisome and the *lacZ* locus during DSBR. (A)** Distribution of the duration of YPet-DnaN foci per replication cycle under slow-growth conditions. **(B)** Mean duration of chromosomal replication under slow-growth conditions. Error bars represent SD. **(C)** Replication and segregation of the *lacZ* locus in the absence and presence of DSB induction at the *lacZ* locus. The dashed lines represent extrapolation of the median values for the “NO DSB” and “DSB” data. **(D)** Localization of the *lacZ* locus (LacI-Cerulean) and the replication site (YPet-DnaN) during formation and repair of the replication-dependent DSB. For all the kymographs, the dash lines represent the midcell. The black arrows represent the formation of the transient RecA-mCherry focus at the site of the DSB. The black circles represent colocalization of the LacI-Cerulean focus with the YPet-DnaN focus. Each black circle containing a black arrow represents the colocalization of the LacI-Cerulean focus with the YPet-DnaN focus until disassembly of the transient RecA-mCherry focus at the DSB site. Each kymograph shows a representative cell and was compiled from the phase-contrast and fluorescence (LacI-Cerulean or YPet-DnaN) images of a cell acquired at 1-min intervals. Heat maps are shown as colored bars, where 0 represents background fluorescence within the cell and 1 represents the maximum fluorescence of fluorescent foci. **(E)** Phase-contrast and fluorescence (LacI-Cerulean and YPet-DnaN) images acquired at 1-min intervals were used to estimate the duration between colocalization of a YPet-DnaN focus with a LacI-Cerulean focus and formation of the RecA-mCherry focus at the site of the DSB. For cells with multiple colocalizations (between LacI-Cerulean and YPet-DnaN foci) preceding the formation of the RecA-mCherry focus, the last colocalization event was chosen.

The distribution of durations of the YPet-DnaN foci per replication cycle was not greatly affected by repair of the DSB at the *lacZ* locus (Fig. 4 A), indicating that repair of the palindrome-induced DSB had minimal effect, if any, on the time required to replicate the entire chromosome (mean of 69 ± 8 min in the absence of DSBR and 68 ± 9 min in the presence of DSBR; Fig. 4 B). These observations are in accordance with our expectation that the DSB is generated behind the progressing replication fork at the *lacZ* locus (Eykelenboom et al., 2008). On the assumption that replication occurs at an approximately equal rate around the chromosome as has been demonstrated (Skovgaard et al., 2011), the time taken to replicate the chromosome predicts that the *lacZ* locus is replicated at 32 min after initiation of replication. The median time required for segregation of the sister *lacZ* loci after initiation of replication (formation of a YPet-DnaN focus in newborn cells) was 50 min in the absence of DSB induction (Fig. 4 C), arguing that cohesion of sister chromosomes at the *lacZ* locus lasts a median period of 18 min. The median period between initiation of replication and the separation of *lacZ* loci was 56 min in cells undergoing DSBR (Fig. 4 C), suggesting that DSBR extends sister chromosome cohesion by 6 min, to give a duration of 24 min.

Analysis of the data obtained by time-lapse microscopy also revealed the relationship between DNA replication and DSBR at the *lacZ* locus (Fig. 4 D). We determined that the formation of a RecA-mCherry focus occurred at a median duration of 2.5 min after the colocalization of YPet-DnaN focus with the *lacZ* locus, which indicates the likely time of *lacZ* replication (Fig. 4, D and E). The YPet-DnaN focus did not always remain localized at the midcell after the formation of the RecA-mCherry focus at the site of the DSB (second YPet-DnaN kymograph in Fig. 4 D). This observation demonstrates that the constraint on the dynamic movement of the *lacZ* locus after the formation and disassembly of the RecA-mCherry focus, before *lacZ* focus segregation, is independent of the spatial localization of the replisome and is likely to be local to the site of DSBR. The period of 2.5 min between DNA replication and RecA-mCherry focus formation, added to the 1.5 min median duration of the RecA-mCherry focus (Fig. 2 D) and the 18 min between disassembly of the RecA-mCherry focus and *lacZ* focus splitting (Fig. 2 C), sums to a total of 22 min of cohesion in the cells undergoing DSBR, consistent with the independent measure of 24 min of total cohesion (Fig. 4 C).

We have shown that RecA forms a distinct and transient focus at the site of a replication-dependent DSB induced at the *lacZ* locus of the *E. coli* chromosome. The transient focus was disassembled before segregation of the sister loci. After disassembly of RecA, the recombining loci remained centrally located in the cell and showed reduced mobility, consistent with local constraints that might include the resolution of the DNA structures such as the Holliday junctions generated by the action of RecA. The RecA focus did not mature into an elongated or bundle structure as reported previously, when rare-cutting endonuclease systems were used to generate a site-specific DSB (Lesterlin et al., 2014; Badrinarayanan et al., 2015). RecA-GFP also formed elongated structures during repair of DSBs generated using mitomycin C (Kidane and Graumann, 2005) and UV irradiation (Renzette et al., 2005; Centore and Sandler, 2007), despite these treatments being predicted to produce replication-dependent DSBs. These studies reported that the formation, maturation, and disassembly of the RecA filaments or bundles lasted for ≥45 min during DSBR (Kidane and Graumann, 2005; Lesterlin et al., 2014; Badrinarayanan et al., 2015). In contrast, our data indicate that a single, site-specific, replication-dependent DSB can be repaired much more efficiently. We conclude that a rapid and local mechanism has evolved to repair replication-dependent DSBs by homologous recombination, taking advantage of the proximity of the intact sister chromosome and the consequent facilitation of DNA homology searching.

## Materials and methods

### Bacterial strains and growth conditions

All the *E. coli* strains used in this study are derivatives of BW27784 (Khlebnikov et al., 2001) and are described in Table S1. This background strain enables homogenous expression of the SbcCD endonuclease from the arabinose-inducible promoter. Mutations were introduced by plasmid-mediated gene replacement (PMGR; Merlin et al., 2002) and confirmed by sequencing and spot-test assays, where applicable. All plasmids used for introducing the mutations are derivatives of pTOF24 (Table S2). Sequences of the oligonucleotides that were used for constructing the pTOF24 derivatives and sequencing of the tet operator (*tetO*) and lac operator (*lacO*) arrays are listed in Table S3.

For all microscopy experiments, the *E. coli* strain of interest was grown overnight at 37°C in M9-minimal medium supplemented with 0.2% of glycerol. The M9–glycerol medium was further supplemented with 100 ng/ml of anhydrotetracycline when strains were grown that contained the *tetO* array and expressed the TetR-YPet protein (Possoz et al., 2006). The overnight culture was diluted to an OD_600_ = 0.09 and grown for 3 h (OD_600_ = 0.3–0.35). The bacterial culture was diluted again in the same medium, and 0.2% of arabinose was added for induction of the expression of the SbcCD endonuclease. The diluted culture was further grown for 2 h before microscopy. These conditions were also used for growing *E. coli* cells during analysis of DNA content by flow cytometry. For the spot-test assay, cells were grown overnight in Luria-Bertani (LB) medium before spotting of the cultures on LB agar plates.

### Spot-test assay

A colony of the *E. coli* strain of interest was grown overnight in liquid LB medium at 37°C. The OD_600_ of the overnight culture was adjusted to 1.0 and serially diluted 10-fold. Aliquots of 4 µl of the serially diluted cultures were spotted onto LB agar plates containing either 0.2% arabinose or 0.5% glucose. The LB agar plates were incubated overnight at 37°C.

### Microscopy and image analysis

Conventional widefield fluorescence microscopy was performed with a Zeiss Axiovert 200 fluorescence microscope equipped with a 100× 1.4 NA oil Plan Apochromat objective (phase or differential interference contrast [DIC]), dual OptoLED light source (Cairn Research), an MS-2000 Piezo Z-Stage (Applied Scientific Instrumentation), and an Evolve 512 electron-multiplying charge-coupled device camera (Photometrics). With the exception of time-lapse imaging of the WT and Δ*recX*, Δ*uvrD*, and Δ*dinI* mutants shown in Fig. 2 D, which were acquired with the DIC objective, all the other images were acquired with the phase objective. The microscope was enclosed in an incubation chamber to control the temperature during live-cell fluorescence imaging. Before either snapshot or time-lapse microscopy, the incubation chamber of the microscope was kept at 37°C, which was the temperature at which the cells were grown in the liquid M9–glycerol medium.

Molten agarose (1.5%) was prepared in M9–glycerol medium supplemented with arabinose and was mounted within a 1.5 × 1.6–cm Gene Frame (Thermo Fisher Scientific), which was sealed onto a microscope slide. A 6-µl aliquot of the cells growing in M9–glycerol medium was spread onto the solidified agarose and sealed with a cover slide. The MetaMorph software (Molecular Devices) was used for image acquisition. Phase-contrast or DIC images were acquired concurrently with the fluorescence images. For snapshot microscopy, each fluorescence image consisted of 11 z sections with 200 nm z distance. The fluorescence images that were acquired during time-lapse microscopy consisted of 6 z-sections with 350nm z-distance to minimize potential photobleaching. The following settings were used during image acquisition: an exposure time of 50 ms and a gain of one for phase and DIC; an exposure time of 50 ms and a gain of 150 for RecA-mCherry; and an exposure time of 100 ms and a gain of 250 for LacI-Cerulean, TetR-Ypet, and DnaN-YPet images.

The images obtained from microscopy were analyzed with either ImageJ (National Institutes of Health) or MetaMorph software. During analysis, the z sections of each fluorescence image were converted to a single image that corresponded with the sum of the stacked images. The sum image was deconvolved with *AutoQuant* X software (Media Cybernetics). Deconvolution was performed to reduce noise and improve the resolution of the fluorescence image. Colocalization analysis was performed with the MetaMorph software and ImageJ (0.4 µm was used as the upper limit for the distance between centroids of the two foci under consideration). The OUFTI software (Paintdakhi et al., 2016) was used for generating kymographs of the images that were obtained from time-lapse microscopy. OUFTI software was also used for measuring cell lengths and distances between the centroid of the LacI-Cerulean foci and an arbitrary cell pole or the midcell (in AU). In all experiments the AU are the same. The cftool function of MATLAB (MathWorks) was used for fitting Gaussian curves on the data illustrating distances of LacI-Cerulean foci from the midcell.

### Quantification of cellular position of *lacZ* locus

Time-lapse images were acquired for RecA-mCherry and LacI-Cerulean at time intervals indicated. The time point at which two distinct LacI-Cerulean foci were formed during *lacZ* loci segregation was used as a reference point for determining the cellular position of the LacI-Cerulean foci during cohesion, which was 18 min before the occurrence of segregation (see Results and discussion). The time points preceding the 18-min cohesion were defined as “before cohesion.” The procedure was repeated for predicted durations of cohesion (7, 9, 12, and 15 min, instead of the 18 min that was determined in the absence of DSB induction.

In the presence of DSB induction, appearance of the RecA-mCherry focus was used as the reference point for determining the cellular position of LacI-Cerulean foci before repair and during repair. During-repair data were collated from the appearance of the RecA-mCherry focus until splitting of the LacI-Cerulean focus. This approach was used because disassembly of the RecA-mCherry focus might not signify the end of the DSBR because of the presence of unresolved joint molecules.

### Analysis of DNA content by flow cytometry

Cells in exponential phase of growth (OD_600_ = 0.3–0.35) at 37°C in M9–glycerol medium were treated with cephalexin and rifampicin. Cephalexin and rifampicin were added at a final concentration of 10 and 150 µg/ml, respectively, and the cells were grown for a further 3 h. The overnight, exponential phase and cephalexin/rifampicin-treated (runout) cultures were separately fixed in 70% ethanol and stored overnight at 4°C. The cells in 70% ethanol were harvested and washed in 1× PBS, and the DNA of these cells was stained with 1× propidium iodide solution for 1 h in the dark at room temperature. An A50 Micro Flow Cytometer (Apogee Flow Systems) was used for recording the fluorescence signal generated by the stained cells after excitation with the blue laser (488 nm). The data obtained were analyzed with Apogee Histogram Software (version 3.1).

### Sanger sequencing of DNA

The complete DNA sequences of the *tetO* and *lacO* arrays were determined via Sanger sequencing of PCR products amplified from the chromosomal loci bearing these operator arrays. PCR products were purified using the QIAquick PCR Purification kit (QIAGEN) before sequencing. The Sanger sequencing reaction was set up using the BigDye Terminator v3.1 cycle-sequencing kit (Applied Biosystems), following the manufacturer’s guidelines. The samples were sent to the Edinburgh Genomics Facility for analysis using the ABI 3730XL capillary-sequencing instrument.

### Statistical analysis

The Mood’s median test was used for ascertaining whether there was a statistically significant difference between the median duration of the RecA foci in WT cells compared with the corresponding duration in each deletion mutant that was studied (Δ*uvrD*, Δ*recX*, or Δ*dinI* mutant). The Mood’s median test was chosen for this analysis because the durations of RecA foci were determined independently for each bacterial strain and the distributions of these durations were similar (right-skewed) for all four strains. For each of these bacterial strains, the individual durations of RecA foci that were either above or below the calculated median value (from Fig. 2 D) were counted and used for calculating the χ^2^ statistic. The χ^2^ statistic was calculated for the WT strain and a mutant strain to ascertain whether the difference between the median durations of RecA foci for these two strains were statistically significant. This procedure for calculating the χ^2^ statistic was separately performed for each of the three mutants in comparison with the WT strain.

### Online supplemental material

Figs. S1 and S2 confirm that DSBR and not postreplicative cohesion localizes the *lacZ* locus in the midcell region. Fig. S3 shows the localization of the replisome in a cell during DNA replication. The *E. coli* strains, plasmids, and oligonucleotides used in this study are provided in Tables S1, S2, and S3, respectively.

## Supplementary Material

Supplemental Materials (PDF)
