# Supplementary material for: Dynamics of RecA-mediated repair of replication-dependent DNA breaks
Source: J Cell Biol. 2018 Jul 2;217(7):2299–307. doi: 10.1083/jcb.201803020 (PMC6028544; doi:10.1083/jcb.201803020)
Supplement: Supplemental Materials (PDF) [file JCB_201803020_sm.pdf]

Supplemental material

Amarh et al., https://doi.org/10.1083/jcb.201803020

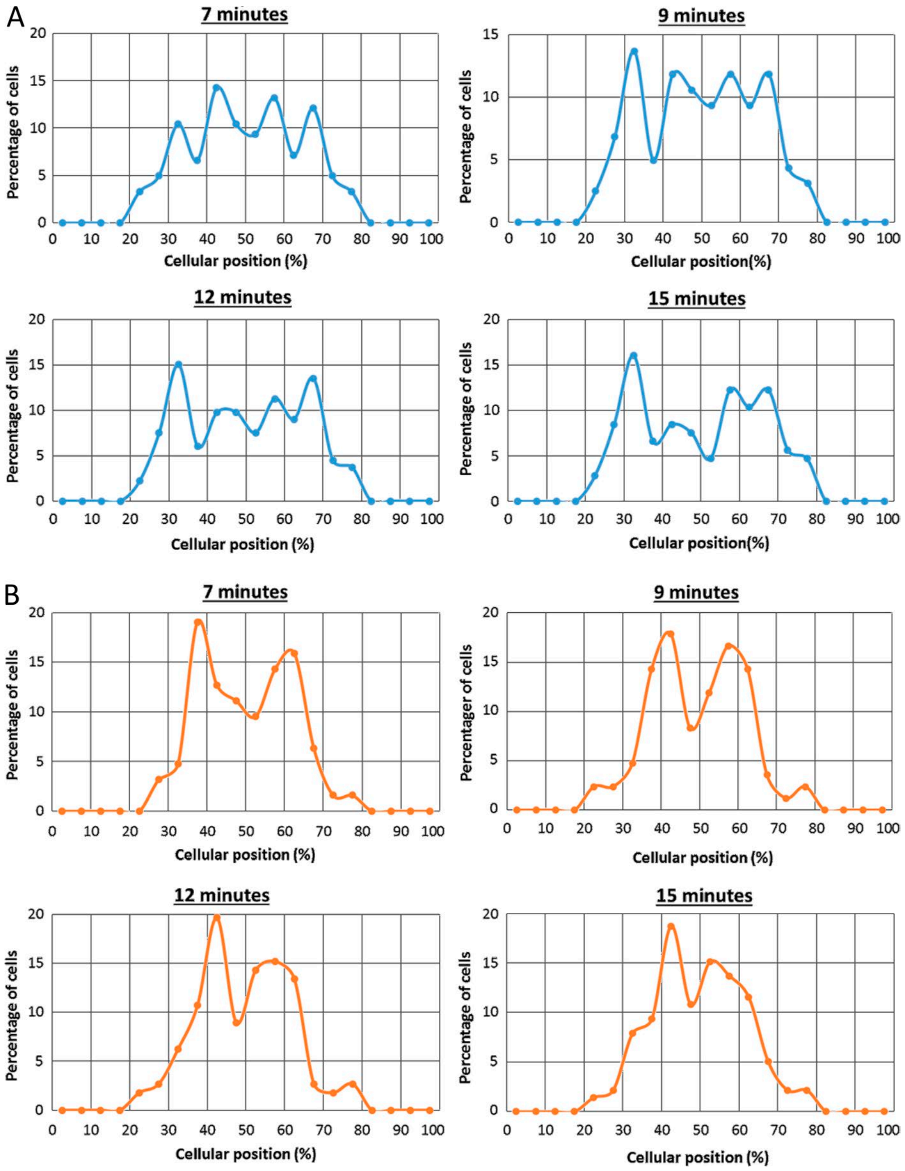

Figure S1. **Localization of the *lacZ* locus before segregation of the sister loci in the absence of DSB induction. (A and B)** In Fig. 3 C, we estimated the effect of postreplicative cohesion on the positions of *lacZ* foci in cells not undergoing DSBR on the assumption that, after DNA replication, the loci stay cohered for a mean of 18 min. This assumption is based on our estimate of 18 min postreplicative cohesion. The locations of foci before the last 18 min before focus splitting are expected to be dominated by *lacZ* loci at prereplicative positions and the locations of foci in the last 18 min before splitting are expected to be dominated by *lacZ* loci in postreplicative cohesion. We tested that assumption by arbitrarily shortening the period of assumed postreplicative cohesion from 18 min to 15, 12, 9, and 7 min and predicted an increase in the contribution of postreplicative cohesion in the distributions. It can be seen that two positions of centralized foci increasingly contributed to the distributions of foci in cells before the period before splitting because it is shortened from 18 to 7 min. The centralized positions dominate at all estimated periods before splitting, but the outside shoulders, which are clearly visible at 18 min (Fig. 3 C), are reduced at shorter times. These data indicate that in the absence of induced DSBR, *lacZ* loci before DNA replication adopt positions centered around one third and two thirds of the cell length and then relocate to two positions within the central 20% of the cell. In contrast, cells undergoing induced DSBR relocate from the one-third and two-third positions to one central location after the formation of a RecA focus, which is taken as a marker separating most prereplicative *lacZ* foci from postreplicative cohered *lacZ* foci (Fig. 3 D). **(A)** Localization of the LacI-Cerulean focus before the arbitrary periods of postreplicative cohesion (*n* = 182 for 7 min, *n* = 161 for 9 min, *n* = 133 for 12 min, and *n* = 106 for 15 min). **(B)** Localization of the LacI-Cerulean focus during arbitrary periods of postreplicative cohesion (*n* = 63 for 7 min, *n* = 84 for 9 min, *n* = 112 for 12 min, and *n* = 139 for 15 min). The indicated time on each graph represents an arbitrary duration of postreplicative cohesion that was chosen for the analysis.

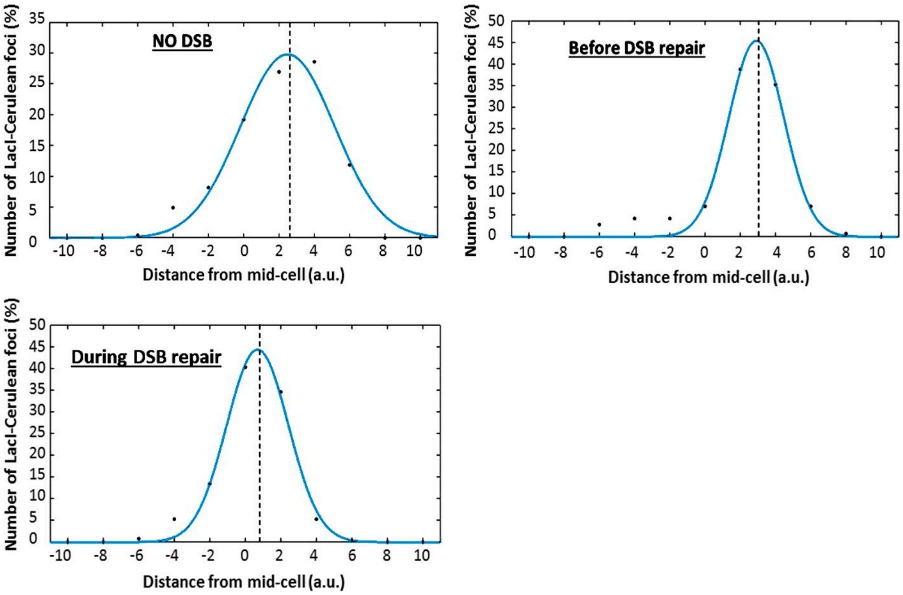

Figure S2. **Effect of DSBR on localization of the *lacZ* locus.** Localization of the LacI-Cerulean focus, in relation to the midcell, in the absence and presence of DSBR. Dash lines represent the peak of the fitted Gaussian curves (in blue). NO DSB was obtained from the strain that did not contain the interrupted palindrome at the *lacZ* locus. Before DSBR was obtained from the palindrome-containing strain before formation of the RecA-mCherry focus at the site of the DSB. During DSBR was obtained from the palindrome-containing strain after the formation of the RecA-mCherry focus until *lacZ* loci segregation (n = 245 for NO DSB, n = 142 for Before Repair, and n = 246 for During Repair). AU used for NO DSB, Before DSBR, and During DSBR were the same.

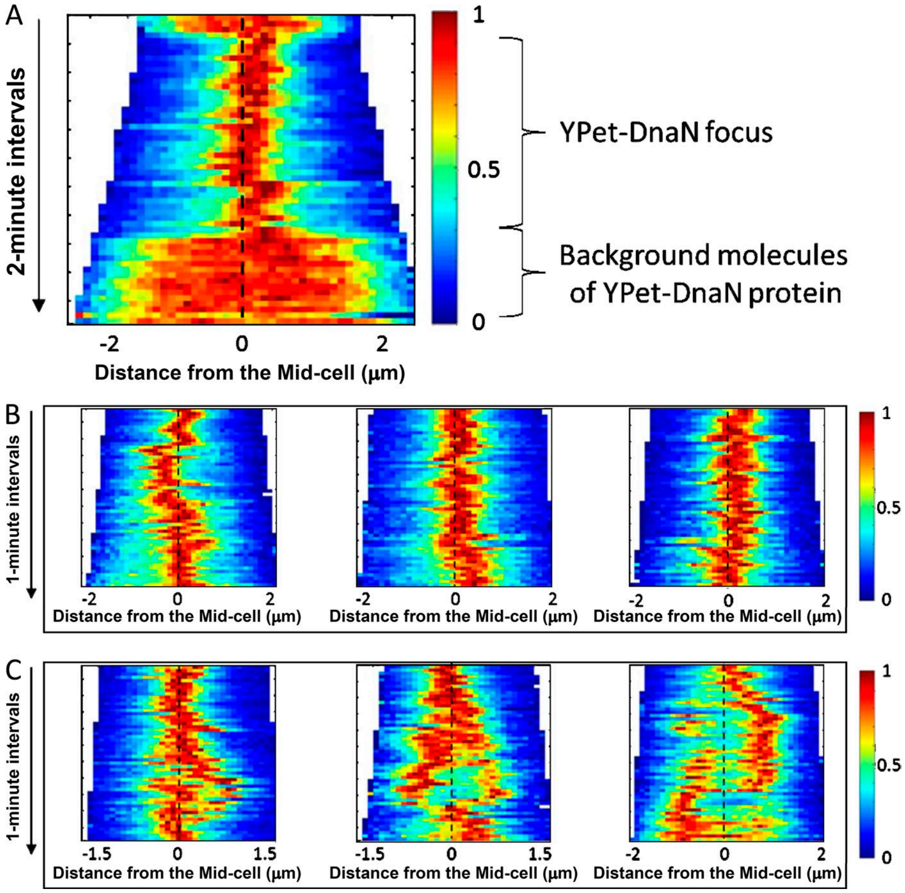

Figure S3. **Dynamics of the foci formed by YPet-DnaN in a cell at exponential phase of growth. (A)** Kymograph of a representative cell showing assembly and disassembly of a YPet-DnaN focus. **(B)** Dynamics of the replisome predominantly localized at the midcell (28 of 41 cells). **(C)** Dynamics of the replisome undergoing separation and remerging during replication (13 of 41 cells). Each kymograph shows a representative cell and was compiled from the phase-contrast and fluorescence (YPet-DnaN) images of a cell acquired at 2-min intervals (A) or 1-min intervals (B and C). Heat maps are shown as colored bars in A, B, and C, where 0 represents background fluorescence within the cell, and 1 represents the maximum fluorescence of YPet-DnaN foci.

Table S1. ***E. coli* strains**

Strain	Relevant genotype	Source
DL3276	P_{araBAD}-*sbcDC lacZ*::pal246 *cynX*::[171x*tetO*(Gm^R)] *mhpC*::[143×*lacO*(Km^R)] Δ*lacI lacZχ- mhpA*::*χχχ lacZY*::*χχχ*	White et al., 2008
DL3277	P_{araBAD}-*sbcDC lacZ*⁺ *cynX*::[171x*tetO*(Gm^R)] *mhpC*::[143×*lacO*(Km^R)] Δ*lacI lacZχ- mhpA*::*χχχ lacZY*::*χχχ*	White et al., 2008
DL5516	*rph*⁺ *ykgC*::P_{mw1}-*lacI-cerulean,tetR-ypet* P_{araBAD}-*sbcDC lacZ*::pal246 *cynX*::[171x*tetO*(Gm^R)] *mhpC*::[143×*lacO*(Km^R)] Δ*lacI lacZχ- mhpA*::*χχχ lacZY*::*χχχ*	DL3276 PMGR using: pDL4068, pDL4522, pDL4680, pDL4690, and pDL5146
DL5521	*rph*⁺ *ykgC*::P_{mw1}-*lacI-cerulean,tetR-ypet* P_{araBAD}-*sbcDC lacZ*⁺ *cynX*::[171x*tetO*(Gm^R)] *mhpC*::[143x*lacO*(Km^R)] Δ*lacI lacZχ- mhpA*::*χχχ lacZY*::*χχχ*	DL3276 PMGR using: pDL4068, pDL4522, pDL4680, pDL4690, and pDL5146
DL5525	DL5521 *recA-recX*::*recAmwg-mCherry*	PMGR using pDL5196
DL5528	DL5516 *recA-recX*::*recAmwg-mCherry*	PMGR using pDL5196
DL5865	DL5528 Δ*uvrD*	PMGR using pDL2391
DL5866	DL5528 Δ*recA*	PMGR using pDL5855
DL5901	DL5525 Δ*uvrD*	PMGR using pDL2391
DL5924	DL5521 Δ*recA*	PMGR using pDL2711
DL5941	DL5516 Δ*recA*	PMGR using pDL2711
DL5942	DL5525 Δ*recA*	PMGR using pDL5855
DL6107	DL5528 Δ*lacI-cerulean* Δ*tetR-ypet*	PMGR using pDL2802
DL6123	DL6107 ykgC::P_{mw1}-*lacI-cerulean*	PMGR using pDL4580
DL6155	DL5525 Δ*recX*	PMGR using pDL6140
DL6156	DL5528 Δ*recX*	PMGR using pDL6140
DL6306	DL6123 *YPet-dnaN*	PMGR using pDL6255
DL6312	DL6123 *lacZ*⁺	PMGR using pDL1823
DL6326	DL6312 *YPet-dnaN*	PMGR using pDL6255
DL6446	DL5528 Δ*dinI*	PMGR using pDL4423
DL6465	DL5525 Δ*dinI*	PMGR using pDL4423

lacZ::pal246 represents the insertion of a 246-bp interrupted palindrome at the *lacZ* locus; *χχχ* represents an array of three Chi sites inserted at the *mhpA* and *lacZY* loci. *lacZY* represents the intergenic region between *lacZ* and *lacY*. *lacZχ-* represents an allele of *lacZ* containing a deletion of one endogenous Chi site. Gm^R, gentamycin resistance (10 µg/ml); Km^R, kanamycin resistance (50 µg/ml).

Table S2. **Plasmids**

Plasmid	Description	Source
pDL1605	pTOF$_{24}$ plasmid [Cm^R Km^R Ts Suc^S]	Merlin et al., 2002
pDL1823	pTOF$_{24}$ derivative for converting *lacZ*::pal246 to *lacZ*$^+$	Zahra et al., 2007
pDL2391	pTOF$_{24}$ derivative for generating Δ*uvrD* mutation	Blackwood et al., 2010
pDL2711	pTOF$_{24}$ derivative for generating Δ*recA* in strains without the *recA-mCherry* gene integrated at the *recA-recX* intergenic region.	This study
pDL2802	pTOF$_{24}$ derivative for generating Δ*lacI-cerulean* and Δ*tetR-ypet* in strains with these genes integrated into the *ykgC* locus	This study
pDL4068	pTOF$_{24}$ derivative for integrating P$_{mw1}$-*lacI-cerulean* and *tetR-eyfp* into the *ykgC* locus	This study
pDL4423	pTOF$_{24}$ derivative for generating Δ*dinI* mutation	This study
pDL4522	pTOF$_{24}$ derivative for converting *hupA*$^+$ to *hupA-mCherry*	This study
pDL4580	pTOF$_{24}$ derivative for integrating P$_{mw1}$-*lacI-cerulean* into the *ykgC* locus	This study
pDL4680	pTOF$_{24}$ derivative for converting *tetR-eyfp* to *tetR-ypet* in strains containing the fluorescence protein at the *ykgC* locus	This study
pDL4690	pTOF$_{24}$ derivative for converting a *rph*$^-$ strain to a *rph*$^+$ strain	Cockram et al., 2015
pDL5146	pTOF$_{24}$ derivative for converting *hupA-mCherry* to *hupA*$^+$	This study
pDL5196	pTOF$_{24}$ derivative for integrating *recA-mCherry* between the endogenous *recA* and *recX* genes	This study
pDL5855	pTOF$_{24}$ derivative for deleting the endogenous *recA* gene in strains containing the *recA-mCherry* gene at the *recA-recX* intergenic region	This study
pDL6140	pTOF$_{24}$ derivative for generating Δ*recX* in strains containing the *recA-mCherry* gene at the *recA-recX* intergenic region	This study
pDL6255	pTOF$_{24}$ plasmid for inserting a yp*et* gene and a 33bp DNA linker upstream of the ATG of the endogenous *dnaN* gene	This study

Cm^R, chloramphenicol resistance (50 µg/ml); Km^R, kanamycin resistance (50 µg/ml); Ts, temperature-sensitive origin of replication (grows at 30°C); Suc^s, sucrose sensitive (5% wt/vol).

Table S3. **Oligonucleotides**

Name	Sequence (5′–3′)	Purpose
DinI.F1	AAAAACTGCAGCATCGCAAAGAGAGCAGTTG	Confirm Δ*dinI* mutation by pDL4423
DinI.R2	AAAAAGTCGACGTGCTGATGTCCCACCCTAC	
Ex-test_F	TTATGCTTCCGGCTCGTATG	Confirm presence or absence of the palindrome at *lacZ*
Ex-test_R	GGCGATTAAGTTGGGTAACG	
HupA-mCherry_F2	AAAAACTCGAGCCCGTCTGGTCTACATTTGG	Confirm the conversion of *hupA-mCherry* to *hupA*⁺
HupA-mCherry_R2	AAAAAGCGGCCGCTGGTCGTTAGAAAGCTGCTG	
RecA-KO-F1	AAAAACTGCAGAACGCGGATTTGTCACCTAC	Confirm Δ*recA* mutation by pDL2711
RecA-KO-R2	AAAAAGTCGACCGCGGGAAATACCTTTCTG	
RecA-mCherry_F1	AAAAAGCGGCCGCCGCTGAAATTCTACGCCTCT	Generating *recA-mCherry* fragment for pDL5196
RecA-mCherry_R1	GGTGAATTCCTCCTGCTAGATTAAAAATCTTCGTTAGT	
RecA-mCherry_F2	TCTAGCAGGAGGAATTCACCATGGCTATCGACGAGAACAA	
RecA-mCherry_R2	CCCTTGGAAACGATGCTCCCAAAGTCCTCGTTGGTTTCGG	
RecA-mCherry_F3	ACGAGGACTTTGGGAGCATCGTTTCCAAGGGCGAGGAGGA	
RecA-mCherry_R3	TTGTGTATCAAACAAGACGATTATTTGTAGAGCTCATCCA	
RecA-mCherry_F4	TGGATGAGCTCTACAAATAATCGTCTTGTTTGATACACAA	
RecA-mCherry_R4	AAAAACTCGAGATTCACGCATCGCTTTTTCT	
RecX2.F1	AAAAACTGCAGCGCTGAAAGGCGAAATAAAA	Generating a Δ*recX* fragment for pDL6140
RecX.R1	AACGCTGGATCTGTCGCAGTTCTTGCTCACTG	
RecX.F2	AAGAACTGCGACAGATCCAGCGTTTTCTGCTC	
RecX.R2	AAAAAGTCGACGTAGCGCGGGAATAATTACG	
Rph-F1	AAAAAGTCGACACAATGGAAATCCAGCGTCT	Generating *rph+* fragment for pDL4690
Rph-R1	CAATGGATTCGATTCCCCCTCGGGCCAGAGC	
Rph-F2	GCTCTGGCCCGAGGGGGAATCGAATCCATTG	
Rph-R2	TTTTTCTGCAGTGCTTCTTTGCGGTTAAAG	
uvrD.F1	AAAACTGCAGTGACCTCGCTGATATAATCA	Confirm Δ*uvrD* mutation by pDL2391
uvrD.R2	AAAAGTCGACTCAGATACTGAAGATGGCGC	
ykgC-F1	AAAAACTGCAGGCATCAACAAACGGCTAAGG	Confirm presence of the DNA insert at the *ykgC* locus
ykgC-R2	AAAAAGTCGACTGTTCTGGCGTCTGATTTTG	
ΔrecA-mCherry F1	AAAAACTGCAGGGTGAGTGAACCCGTCGT	Obtaining a Δ*recA* fragment for pDL5855
ΔrecA-mCherry R1	AAAAATCTTCGTTAGTTTCTTTGTTTTCGTCGATAGCC	
ΔrecA-mCherry F2	GGCTATCGACGAAAACAAAGAAACTAACGAAGATTTTT	
ΔrecA-mCherry R2	AAAAAGTCGACCTGGGAACACAGGAGGTTGT	
YPet-dnaN F1	AAAAACTGCAGAACGTACGTGAGCTGGAAGG	For constructing pDL6255
Same RBS R1	GAATATCCTCCTTAGAAGCAGCTCCAGCCTACAAAGGTTTAC GATGACAATGTTCTGATTAAA	
Same RBS F2	TTGTAGGCTGGAGCTGCTTCTAAGGAGGATATTCATGTCTAA AGGTGAAGAATTATTCACTG	
YPet-dnaN R2	AAAAAGTCGACTTCAATCAGACGCTTCATCG	
seq_tetO-1.F	CAGTGATAGAGAAGACGAACCG	For sequencing the *tetO* array
seq_tetO-1.R	GTAGGGACCATTGCTAGAGTTG	
seq_tetO-2.F	GTAATCGCTCTAGCAAGCG	
seq_tetO-2.R	CTGCTAGAGCCAGCTCG	
seq_tetO-3.F	CAACTCTAGCAATGGTCCCTAC	
seq_tetO-3.R	CATGCTAGAGCCTCCTTAC	
seq_tetO-4.F	CGAGCTGGCTCTAGCAG	

Table S3. **Oligonucleotides *(Continued)***

Name	Sequence (5′–3′)	Purpose
seq_tetO-4.R	CTCTCTGCTAGAGTACCTTC	
seq_tetO-5.F	GTAAGGAGGCTCTAGCATG	
seq_tetO-5.R	GCTCCCCTGCTAGAGATTATAAG	
seq_tetO-6.F	GAAGGTACTCTAGCAGA	
seq_tetO-6.R	CTCTATCACTGGTAGGGACG	
seq_tetO-7.F	CTTATAATCTCTAGCAGGGGAGC	
seq_tetO-7.R	CGTTTGGCCCTGCTAGAG	
seq_tetO-8.F	CGTCCCTACCAGTGATAGAG	
seq_tetO-8.R	CCTTCTGCTAGAGCTACTATTC	
seq_tetO-9.F	CTCTAGCAGGGCCAAACG	
seq_tetO-9.R	GACAGCTACCACTGCTAG	
seq_tetO-10.F	GAATAGTAGCTCTAGCAGAAGG	
seq_tetO-10.R	GTTTCGTTGCTAGAGCACTAG	
seq_tetO-11.F	CTAGCAGTGGTAGCTGTC	
seq_tetO-11.R	CTCGCTCTCTTGCTAGAGTG	
seq_tetO-12.F	CTAGTGCTCTAGCAACGAAAC	
seq_tetO-12.R	CTCTATCACGATAGGGAAGTGC	
seq_tetO-13.F	CACGTGATAGAGATGGAGTTGGA	
seq_tetO-13.R	CTATCACTGATAGGGCCTC	
seq_tetO-14.F	CAGAGGCTCTAGCAGAGAC	
seq_tetO-14.R	CTCTACCACTGATAGGGAAC	
seq_tetO-15.F	GAGGCCCTATCAGTGATAG	
seq_tetO-15.R	CTGCTTGCTAGAGCTGG	
seq_tetO-16.F	GTTCCCTATCAGTGGTAGAG	
seq_tetO-16.R	CTCTATCACTGATAGGAGGCC	
seq_tetO-17.F	CCAGCTCTAGCAAGCAG	
seq_tetO-17.R	CGTCCTTCTCTATCACTGAGTC	
seq_tetO-18.F	GGCCTCCTATCAGTGATAGAG	
seq_tetO-18.R	CGCACTTCTGCTAGAGTTCAAACG	
seq_tetO-19.F	GACTCAGTGATAGAGAAGGACG	
seq_tetO-19.R	CCTCATGCTTGCTAGAGATTG	
seq_tetO-20.F	CGTTTGAACTCTAGCAGAAGTGCG	
seq_tetO-20.R	CTGCTATGCTAGAGTTTAGACC	
seq_tetO-21.F	CAATCTCTAGCAAGCATGAGG	
seq_tetO-21.R	CTTGTCACGATGCTAGAGTC	
seq_tetO-22.F	GGTCTAAACTCTAGCATAGCAG	
seq_tetO-22.R	CTGATAGGGAGATGCACAG	
seq_tetO-23.F	GACTCTAGCATCGTGACAAG	
seq_tetO-23.R	GCTCTCTATCACAGATAGGG	
seq_tetO-24.F	CTGTGCATCTCCCTATCAG	
seq_tetO-24.R	CTGACAGGGACCTCTCTTC	
seq_tetO-25.F	CCCTATCTGTGATAGAGAGC	
seq_tetO-25.R	GTCGACTCTAGAGTACGTC	
Gentamicin 1	AGAGCGTATTACCTTC	For sequencing the gentamycin
Gentamicin 2	GTAGCCACCTACTCCCAACAT	Resistance gene within the *tetO* array

Table S3. **Oligonucleotides *(Continued)***

Name	Sequence (5′–3′)	Purpose
Gentamicin 3	AGATGGGGGCCTCTAGCAA	
seq_lacO-1.F	ACACTTTATGCTTCCGGCTC	For sequencing the *lacO* array
seq_lacO-2.F	ACGGACGGAAAATTGTGAGC	
seq_lacO-3.F	GAACCAGGAACTCTAGCAAG	
seq_lacO-4.F	AGTAGGAAGCTCTAGCAGAC	
seq_lacO-5.F	AGCGGATAACTATTGGGTCG	
seq_lacO-6.F	CGGCTCTAGCATGTTAGCAT	
seq_lacO-7.F	GAAGAGTAGCTCTAGCAAGG	
seq_lacO-8.F	CAAGGAGCTCTAGCAAAAGG	
seq_lacO-9.F	GCACATACCCCGAAATTGTG	
seq_lacO-10.F	GCGGAAACAATTGGGAGACA	
seq_lacO-11.F	CACGCGCATAGGAATTGTGA	
seq_lacO-12.F	GAGCAAAGCTCTAGCACG	
seq_lacO-13.F	TGACCTCTGGAATTGTGAGC	
seq_lacO-14.F	TGATAACAGCGCTCTAGCAG	
seq_lacO-14.R	CGCTCACAATTCCGATATGC	
seq_lacO-15.F	TCTAGCAAAGGTGCCAA	
seq_lacO-15.R	TCTTTCCTTCTGCTAGAGGG	
seq_lacO-16.F	GAATCCAGGACTCTAGCAAC	
seq_lacO-17.F	CCCTCTAGCAGAAGGAAAGA	
seq_lacO-17.R	GTGATGCTAGAGGCTACTTG	
seq_lacO-18.F	CAGTCACTGCTAGAGCATTG	
seq_lacO-19.F	CAAGTAGCCTCTAGCATCAC	
seq_lacO-20.F	ATGGCTCTAGCAATGGAGAG	
seq_lacO-21.F	TGGGGATAAGTCTCTAGCAG	
seq_lacO-22.F	TGGCCAAAAGTCTCTAGCAG	
seq_lacO-23.F	TGAGAAGCGGGAATTGTGAG	
seq_lacO-24.F	TGGCCCCGGATAATTATGAG	
seq_lacO-25.F	AGGCTACGAACTCTAGCAAG	
seq_lacO-26.F	CTCTAGCAGCGGGAAGTTAA	
seq_lacO-27.F	AGGACCTCTAGCAAAGATGG	
seq_lacO-28.F	CAAATGGGCCTCTAGCAATC	
seq_lacO-28.R	TCCGCTCATTTATTACGCCC	
seq_lacO-1.R	CTTGCTAGAGTTCCTGGTTC	
seq_lacO-2.R	GTCTGCTAGAGCTTCCTACT	
seq_lacO-3.R	CGACCCAATAGTTATCCGCT	
seq_lacO-4.R	ATGCTAACATGCTAGAGCCG	
seq_lacO-5.R	CCTTGCTAGAGCTACTCTTC	
seq_lacO-6.R	CCTTTTGCTAGAGCTCCTTG	
seq_lacO-7.R	CACAATTTCGGGGTATGTGC	
seq_lacO-8.R	TGTCTCCCAATTGTTTCCGC	
seq_lacO-9.R	TCACAATTCCTATGCGCGTG	
seq_lacO-10.R	CGTGCTAGAGCTTTGCTC	
seq_lacO-11.R	GCTCACAATTCCAGAGGTCA	
seq_lacO-12.R	CTGCTAGAGCGCTGTTATCA	

https://doi.org/10.1083/jcb.201803020

Table S3. **Oligonucleotides *(Continued)***

Name	Sequence (5′–3′)	Purpose
seq_lacO-13.R	CTGAATTGTTATCCGCTCGC	
seq_lacO-16.R	AATGCTCTAGCAGTGACTGG	
seq_lacO-17.R	GTGATGCTAGAGGCTACTTG	
seq_lacO-18.R	CTCTCCATTGCTAGAGCCAT	
seq_lacO-19.R	CTGCTAGAGACTTATCCCCA	
seq_lacO-20.R	CTGCTAGAGACTTTTGGCCA	
seq_lacO-21.R	CTCACAATTCCCGCTTCTCA	
seq_lacO-22.R	CTCATAATTATCCGGGGCCA	
seq_lacO-23.R	CTTGCTAGAGTTCGTAGCCT	
seq_lacO-24.R	TTAACTTCCCGCTGCTAGAG	
seq_lacO-25.R	CCATCTTTGCTAGAGGTCCT	
seq_lacO-26.R	GATTGCTAGAGGCCCATTTG	
seq_lacO-27.R	TGCAGTGCTAGAGTAAACCC	
LacO H1	TTACGCCGATGCCAACCGAA	
LacO H2	CGTTCACCCTTAAATGGCCG	
seq_lacO-29.R	CTCGAATTGTGCTCACAATTG	
LacO H3	GCTAGAGCAATCTTGCCAATTG	
seq_lacO-30.R	GTTATCCGCTCACAATTTCTCC	

The underlined sequences represent the recognition sites of restriction enzymes that were used for constructing the $pTOF_{24}$ plasmid derivatives.

References

Blackwood, J.K., E.A. Okely, R. Zahra, J.K. Eykelenboom, and D.R. Leach. 2010. DNA tandem repeat instability in the *Escherichia coli* chromosome is stimulated by mismatch repair at an adjacent CAG·CTG trinucleotide repeat. *Proc. Natl. Acad. Sci. USA.* 107:22582–22586. https://doi.org/10.1073/pnas.1012906108

Cockram, C.A., M. Filatenkova, V. Danos, M. El Karoui, and D.R. Leach. 2015. Quantitative genomic analysis of RecA protein binding during DNA double-strand break repair reveals RecBCD action in vivo. *Proc. Natl. Acad. Sci. USA.* 112:E4735–E4742. https://doi.org/10.1073/pnas.1424269112

Merlin, C., S. McAteer, and M. Masters. 2002. Tools for characterization of *Escherichia coli* genes of unknown function. *J. Bacteriol.* 184:4573–4581. https://doi.org/10.1128/JB.184.16.4573-4581.2002

White, M.A., J.K. Eykelenboom, M.A. Lopez-Vernaza, E. Wilson, and D.R. Leach. 2008. Non-random segregation of sister chromosomes in *Escherichia coli*. *Nature.* 455:1248–1250. https://doi.org/10.1038/nature07282

Zahra, R., J.K. Blackwood, J. Sales, and D.R. Leach. 2007. Proofreading and secondary structure processing determine the orientation dependence of CAG x CTG trinucleotide repeat instability in *Escherichia coli*. *Genetics.* 176:27–41. https://doi.org/10.1534/genetics.106.069724
